# Laser-treated glass platform for rapid wicking-driven transport and particle separation in bio microfluidics

**DOI:** 10.1039/c9ra03448j

**Published:** 2019-06-21

**Authors:** Hongjie Jiang, Manuel Ochoa, Rahim Rahimi, Wuyang Yu, Babak Ziaie

**Affiliations:** School of Electrical and Computer Engineering, Purdue University West Lafayette IN 47907 USA bziaie@purdue.edu; Birck Nanotechnology Center, Purdue University West Lafayette IN 47907 USA; Weldon School of Biomedical Engineering, Purdue University West Lafayette IN 47907 USA

## Abstract

In this work, we present a laser-based fabrication technique for direct patterning of micro-channels consisting of interconnected micro-cracks on soda-lime glass. Using a CO_2_ laser to deposit energy at a linear rate of 18.75 to 93.75 mJ mm^−1^, we were able to manipulate the micro-crack formation, while enabling rapid manufacturing and scalable production of cracked-glass microfluidic patterns on glass. At the higher end of the energy deposition rate (93.75 mJ mm^−1^), the laser fabricated microfluidic channels (1 mm wide and 20 mm long) had extremely fast wicking speeds (24.2 mm s^−1^, ×10 faster than filter paper) as a result of significant capillary action and laser-induced surface hydrophilization. At the lower end (18.75 mJ mm^−1^), 3–4 μm wide micro-cracked crevices resulted in an increased mesh/sieve density, hence, more efficiently filtering particle-laden liquid samples. The reproducibility tests revealed an averaged wicking speed of 10.6 ± 1.5 mm s^−1^ measured over 21 samples fabricated under similar conditions, similar to that of filter paper (∼85%). The micro-cracked channels exhibited a stable shelf life of at least 82 days with a wicking speed within 10–13 mm s^−1^.

## Abstract 2

In this work, we present a laser-based fabrication technique for direct patterning of micro-channels consisting of interconnected micro-cracks on soda-lime glass.

## Graphical abstract

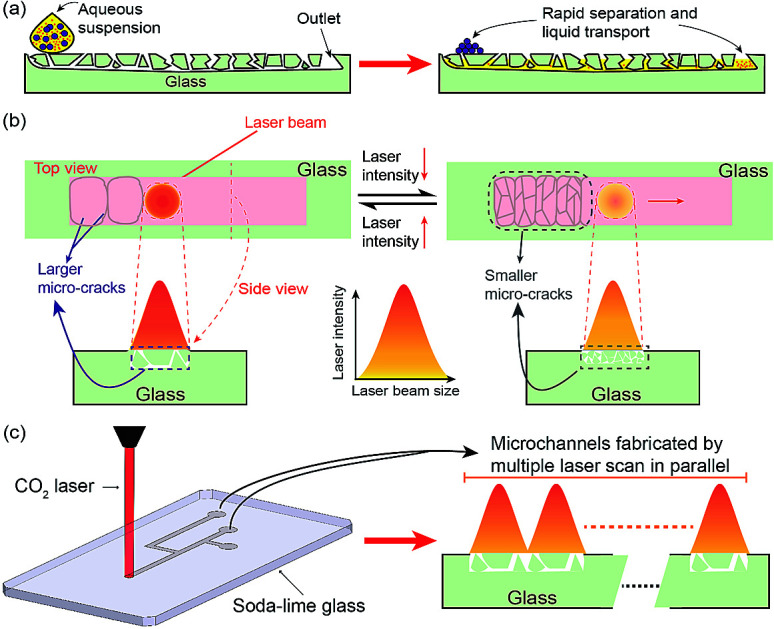

## Introduction

Due to its optical transparency, rigidity, bio-compatibility, and ease of surface modification/functionalization, glass has traditionally been one of the workhorse materials in the fabrication of microfluidic and other biomedical lab-on-a-chip devices.^1–3^ Recently, researchers have investigated lower-cost, flexible substrates such as functionalized polymers^2,4,5^ and paper,^6–8^ whose remarkable inherent wicking properties and filtration capabilities have allowed the realization of a variety of passive analytical microsystem.^8–10^ Wicking, in particular, offers the advantage of passive liquid transport^11,12^ without the need for a micro-pump, thus significantly reducing the system complexity and cost. Similarly, passive filtration *via* a mesh structure provides an inexpensive particle separation method.^13,14^ Together, wicking and particle separation are two principal necessities for many microfluidics and lab-on-a-chip applications, but they often require multiple distinct materials (*e.g.*, paper, polymer filters) which are difficult to integrate with established microfabrication techniques and materials. Imparting such capabilities (*i.e.*, wicking and filtering) to glass would allow for a unique platform that combines the reliability and established surface chemistry of glass with the passive fluidic transport and filtration properties of paper.

As a first step towards this goal, we have developed a facile laser processing method to fabricate glass with wicking properties, rapid liquid transport, and particle separation capabilities. Here, we demonstrate that laser machining can be used to directly inscribe micro-channels comprising of a series of interconnected micro-cracks. Unlike previous works, in which a laser was utilized to remove/etch channels on the glass,^15–20^ our method enables the generation and retention of cracked glass fragments within the channel through precise control of laser parameters, thus imparting wicking properties onto the glass. Moreover, the use of laser for creating the channels offers a scalable manufacturing process that simultaneously allows for fast design iterations. The use of laser machining fills a technological gap between wafer-level micromachining and commercial mass production, with the additional advantage of reducing turn-around time and cost when experimenting with multiple designs (ideal for use in research and development laboratories).

**Fig. 1:**
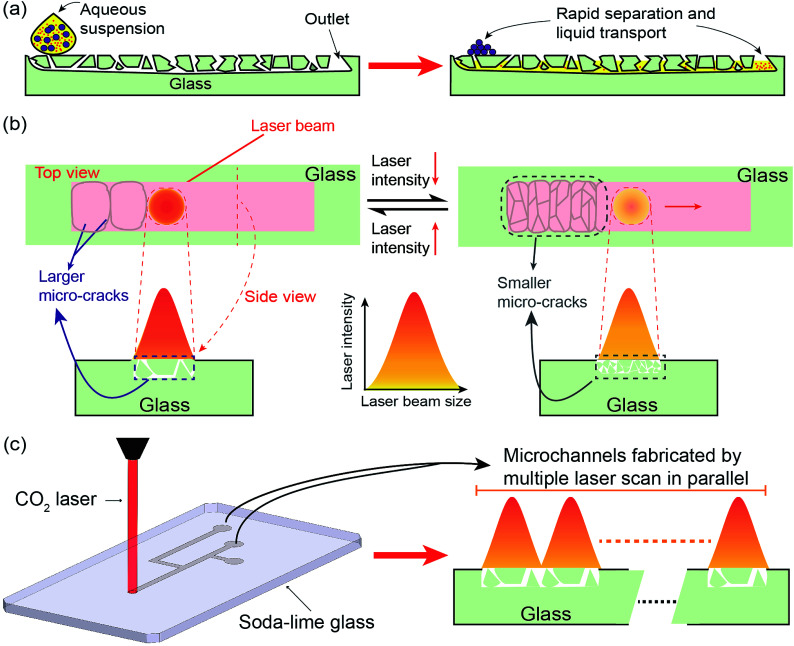
(a) Cross-sectional schematic of the laser-machined glass structure showing its wicking mechanism: (left) an aqueous solution prior to contacting the glass; (right) the solution wicks into the fractured glass and quickly propagates across the channel. (b) The conceptual illustration of the laser ablation of the glass surface showing the relationship between the cracks formation and laser energy deposition rate. Larger micro-cracks at smaller densities, suitable for fast wicking action, forms at higher energies; while smaller and more numerous micro-cracks, suitable for filtering, forms when the energy deposition rate is reduced. (c) Fabrication of a series of the wicking glass microfluidic channels by sequentially scanning the laser beam along the channel length.

Fig. 1(a) illustrates the cross-sectional structure and principle behind the wicking action of a cracked-glass microchannel. The channel is defined by a multitude of inter-connected surface/sub-surface cracks created in a controlled fashion. The cracks comprise each channel feature dimensions no larger than 3–4 μm, allowing for rapid wicking action due to capillary forces; the liquid flow is further aided by the addition of hydrophilic functional groups created by the laser treatment. When an aqueous suspension containing different sizes of nano or micro particles is deposited on one end of the channel, the liquid seeps into the cracks and is drawn across the length of the channel. Any particles larger than the cracks are filtered from the liquid and remain at the beginning of the channel, whereas the smaller particles are able to reach the outlet. In such case, the cracked glass structure achieves a dual function of rapid liquid transportation and particles separation/filtration.

The performance of the laser-processed glass microfluidic platform, including wicking and filtering, relies on the size morphology of the cracked glass, which itself depends on the control of laser–glass interactions. Since glass cracking is ultimately the result of thermos-elastic effects, it can be controlled by tuning/controlling the energy imparted to the substrate by the laser. Interaction of laser radiation with glass can result in direct ablation *via* a sputtering effect (*e.g.*, as with intense pulses using a femtosecond laser)^21–24^ or melting/cracking by photo thermal effects.^25–30^ Infrared (10.6 μm) CO_2_ laser radiation affects the glass through the latter process.^27^ The laser imparts thermal energy onto the glass surface at a rate depending on the laser power level and scanning speed. At the lower ends of energy deposition rate (3.0 J (cm^−2^ s^−1^))^27^ in which the glass reaches its strain point (514 °C for soda lime),^27,31^ thermal effects result in the formation of micro-cracks due to thermal shock, with crack size increasing with energy. At higher energy levels (6.0 J (cm^−2^ s^−1^)),^27^ the glass temperature can reach and surpass the glass softening point (720 °C),^27,31,32^ allowing it to deform or even melt/vaporize and re-deposit to form fewer but longer/deeper cracks. Therefore, both the crack size and density can be simultaneously controlled by tuning the laser energy deposition rate, Fig. 1(b). In the present work, the two types of aforementioned cracks are used to provide different microfluidic capabilities in the channels. Larger cracks are created to produce fast wicking speed by providing more straight wicking paths and reducing unnecessary branching/routing, whereas smaller crack networks are created to provide filtration capabilities (albeit at slower wicking speeds). This allows us to create microchannel networks with controlled transport and filtration properties through simple adjustment of laser energy (power and scanning speed).

## Experimental section

### Fabrication of the cracked-glass microfluidic platform

The fabrication process of the wicking glass microchannel is straightforward and economical, Fig. 1(c). First, a microchannel network is designed in vector graphics software and imported onto the CO_2_ laser engraver system (PLS6MW, Universal Laser Systems, Inc., Scottsdale, AZ). The system then inscribes the pattern on the soda lime glass (GOLD SEAL® Micro Slide) slide through multiple laser scans in parallel, producing cracked glass microchannels. As discussed previously, this process will generate micro-crevices/cracks based on the thermal energy imparted onto the glass surface, whose interconnections form a microfluidic channel enabling capillary action for wicking while simultaneously acting as a mesh for filtering particles.

### Characterization of the cracked patterns

For characterizations of the effect of laser parameters on crack patterns the glass slide was irradiated using a single laser beam scan with the scanning rate set to 0.8 mm ms^−1^ and the laser power set to various levels between 15 to 75 W (at 15 W intervals, resulting in linear energy densities of 18.75 to 93.75 mJ mm^−1^). For each laser beam setting, the crack length (mm) and density (crack per mm) were measured using a calibrated optical microscope. Next, these laser beam configurations were utilized to create a 1 mm wide and 20 mm long microfluidic channel by applying multiple parallel laser scans. The effects of the interconnected micro-cracks on the channel were demonstrated by measuring and comparing the cracks density (cracks per mm^2^) over a 1 mm^2^ laser-processed region at the end of the channel.

### Characterization of wicking and separation/filtration

To characterize the wicking and filtering properties of the platform, an “I” channel (1 mm wide × 20 mm long) was designed and fabricated. It was first used to quantitatively investigate the liquid transport properties of the platform by measuring the average wicking speed of an aqueous dye. The “I” channels were created using an extended laser beam setting, where the laser power was kept to be from 15 to 75 W at 15 W intervals while the scanning speed was adjusted from 0.8 to 4 mm ms^−1^ (linear energy densities of 3.75 to 93.75 mJ mm^−1^). After fabrication, channels were tested immediately by depositing 1 μL of an Evans Blue solution at the inlet of the channel and measuring the average time elapsed until the wicking solution reached the end of the channel using a hand-held stopwatch; the channel length was sufficiently large to allow for reliable precision when starting and stopping the stopwatch.

The “I” channel was then used to qualitatively demonstrate the separation capability of the structure. This was done by depositing aqueous liquid solution containing a set of different size of particles, including silica spheres (9–13 μm), iron oxide micro-particles (<5 μm), and silver nanoparticles (∼200 nm). The liquid was then wicked along the channel and the particles at the inlet and outlet were checked by SEM imaging. Finally, the quantitative filtering performance was characterized by (1) wicking/filtering 1 μL magnetic polystyrene bead solution (magnetic PS) (∼1.5 μm diameter, ProMag, Bangs Laboratories) through the 20 mm long “I” channel; (2) drying the glass and subsequently immersing the PS beads from the inlet (before filtering) and from the outlet (2 mm diameter circle) into 1 ml DI water (each) to form uniform dispersions; (3) measuring and comparing the difference of scattered light intensity between inlet and outlet solutions by using a particle size analyzer (Nano ZS Zetasizer, Malvern) in terms of percentage.

### Characterization of reproducibility and shelf life

Due to the statistical/random nature of the cracking process, it is very difficult to predict the exact crack size and pattern for an individual sample. However, since each channel consists of multiple crack formations due to the actions of the laser beam, it is reasonable to assume a reasonable repeatability/reproducibility across the samples. To test this assumption, we conducted robustness characterizations using 21 samples fabricated under the same laser settings (45 W power and 1.6 mm ms^−1^ scan rate). In addition, we investigated the shelf-life of the device. For this, seven groups of samples (total 21 channels) were fabricated, stored in ambient conditions, and their wicking properties were measured at intervals of ∼3 days for >90 days (day 0 was defined as the day at which the channels were fabricated). The power and scanning speed for these set of devices were also set to 45 W and 1.6 mm ms^−1^.

## Results and discussion

### Micro-cracks pattern

**Fig. 2:**
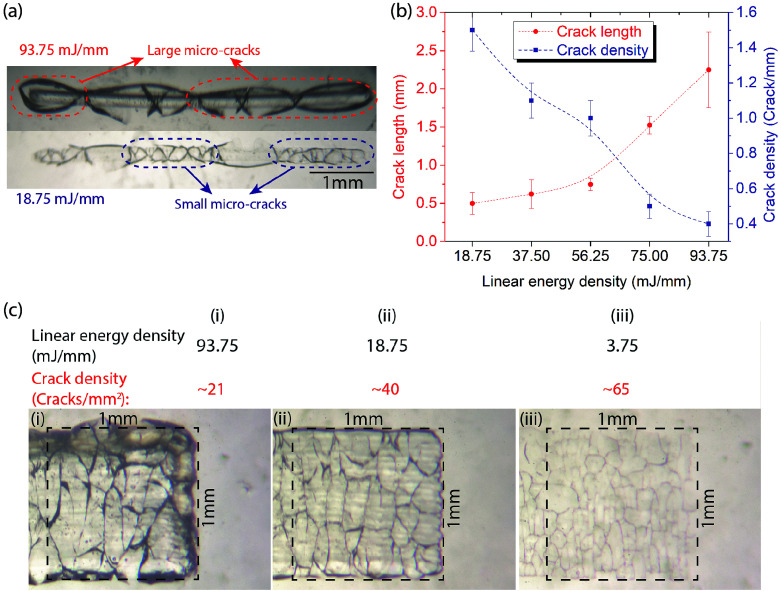
(a) The microscopic images of laser-processed glass (a single scan) under the linear energy density of 93.75 and 18.75 mJ mm^−1^, respectively. When raising the laser intensity, the created micro-cracks have longer length but smaller density (cracks per mm). (b) Crack length and density as a function of laser linear energy density. When applying a linear energy density from 18.75 to 93.75 mJ mm^−1^, the crack length and density are controlled in the range of 0.5–2.25 mm and 1.5–0.4 crack per mm respectively. (c) The microscopic images of a microchannel fabricated by scanning the laser several times along the length of the channel. Within a 1 mm^2^ laser-engineered region, a linear energy density of 93.75 mJ mm^−1^ (i) can create a cracks density of 21 cracks per mm^2^, which would increase to 40 and 65 cracks per mm^2^ by decreasing the density to 18.75 (ii) and 3.75 mJ mm^−1^ (iii) respectively.

Fig. 2(a) presents a comparison of the cracking pattern between two linear energy densities of 18.75 mJ mm^−1^ and 93.75 mJ mm^−1^ using a single beam scan. As can be seen, high laser intensity generates large micro-cracks with concurrently smaller crack density (number of independent cracks per distance or per area), whereas lower laser intensity generated cracks that are smaller and denser. A quantitative analysis of the crack length and density are presented in Fig. 2(b). By increasing the linear energy density from 18.75 to 93.75 mJ mm^−1^, the crack length increases from 0.5 to 2.25 mm while the linear crack density decreases from 1.5 to 0.4 crack per mm. As a result, a channel of 1 mm width created with energy density of 93.75 mJ mm^−1^ has a small crack (area) density of 21 cracks per mm^2^. The crack density is increased to 40 cracks per mm^2^ by decreasing the linear energy density to 18.75 mJ mm^−1^ (this can be increased further to 65 cracks per mm^2^ at energy density of 3.75 mJ mm^−1^), Fig. 2(c). As discussed previously, with larger cracks, the channels are expected to exhibit a faster wicking speed and a lower filtering capability. The reverse (slow wicking but high filtering) is valid for the channels consisting of small cracks. Therefore, one can manipulate the morphology and interaction of the micro-cracks to optimize for the desired liquid transport and particle filtering properties.

### Wicking performance

**Fig. 3:**
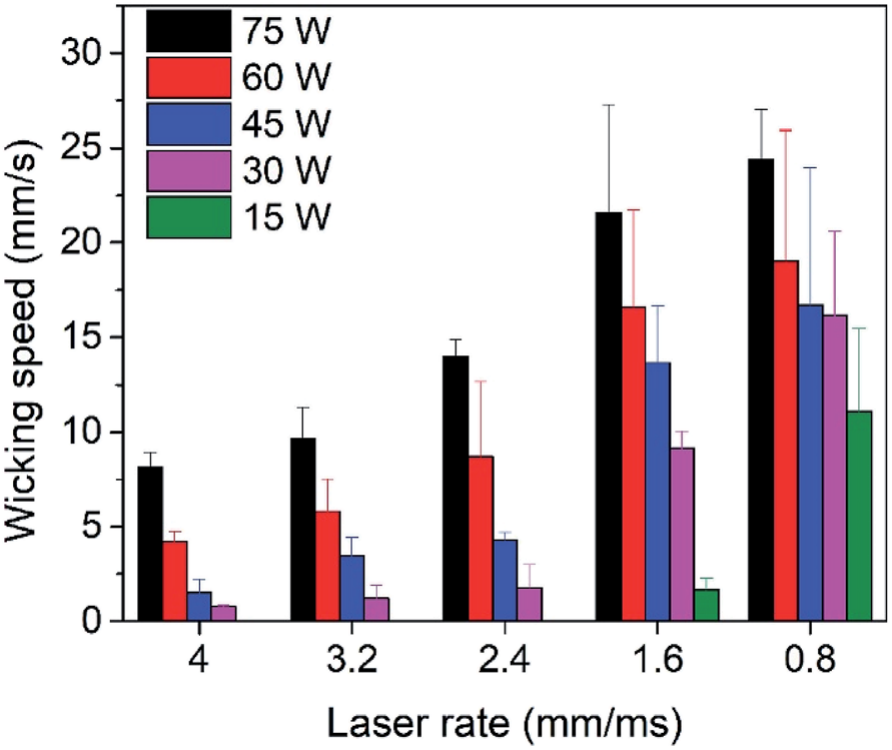
Liquid wicking speed as a function of laser fabrication parameters (power and speed). The wicking speed can be controlled in the range of 0.8–24.2 mm s^−1^. Each data point represents the average of 3 trials.

The wicking performance of the micro-channels was characterized by measuring the average speed to transport an aqueous dye in the “I” channels under various combinations of the laser parameters. The results of the transport speed experiments are plotted in Fig. 3. The data show a strong dependence of wicking velocity on both, the power and processing speed of the laser system (*i.e.*, deposited energy). In particular, the average wicking velocity increases with increasing energy (higher laser power or lower speed). This confirms the proportional relationship between the laser intensity and wicking speed. By using a power of 75 W and processing speed of 0.8 mm ms^−1^, we achieved an average wicking velocity of 24.2 mm s^−1^. This value is significantly faster than the typical 2 mm s^−1^ reported for filter paper,^33^ allowing for liquid transport and particle separation at speeds superior to those of paper microfluidics. Additionally, unlike paper, the wicking velocity of the glass platform is controllable along a wide spectrum of values down to 0.8 mm s^−1^ by varying the laser parameters. This broad range of possible wicking velocities makes the platform suitable for many applications requiring controlled liquid transport.

**Fig. 4:**
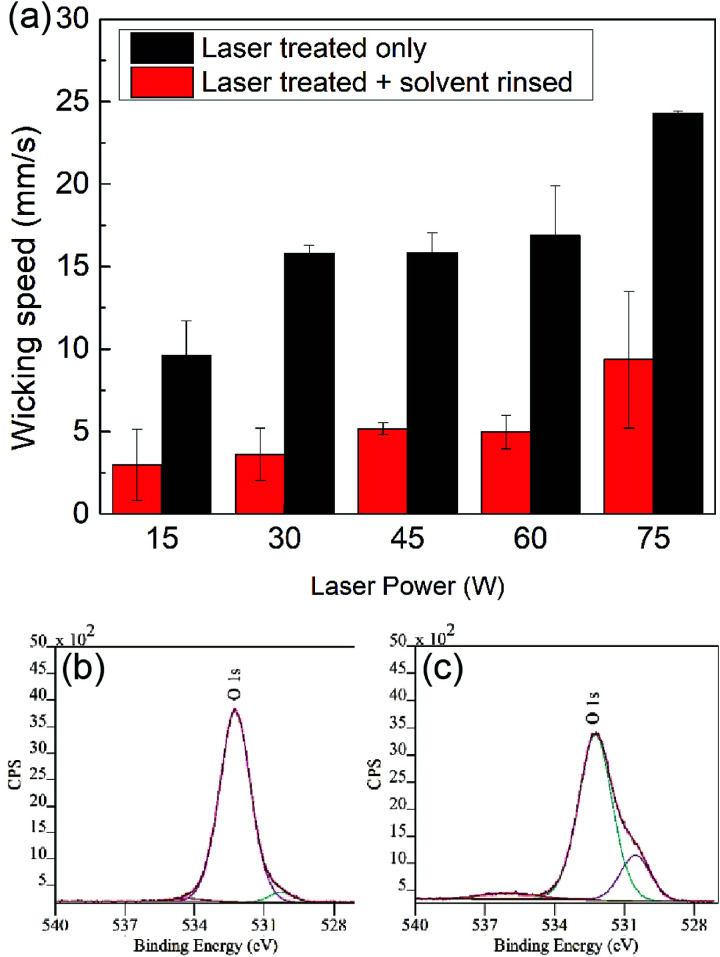
(a) Fluid transport/wicking velocity is diminished when the samples are solvent-cleaned after laser-machining, suggesting the removal of hydrophilic functional groups. For each power setting, the data represent the average of 3 samples, each with a different processing speed of 0.8 mm ms^−1^. XPS data of the O 1s spectra for soda-lime glass (b) before, and (c) after laser treatment. The data suggest an increase in the amount of hydrophilic functional groups (*e.g.*,–OH) on the glass after laser treatment.

As mentioned previously, the high wicking speeds observed in Fig. 3 can be attributed to the increased crack size at high laser intensities (these cracks are also more straight). In addition to this physical phenomena, a chemical factor, *i.e.*, surface hydrophilization also contributes to this behavior. The chemical effect can be observed by comparing the average wicking velocity of an aqueous solution on glass channels just after laser treatment to samples which were solvent (isopropyl alcohol) cleaned after fabrication prior to the test. Here, the solvent cleaning step is expected to promote condensation of surface functional groups generated during fabrication. The results of this comparison are presented in Fig. 4(a), showing a higher overall wicking speeds with a stronger dependence on laser parameters among the untreated samples compared to the solvent-cleaned ones, and thus demonstrating the existence of a laser-induced hydrophilic surface chemistry prior to the solvent cleaning.^34^

To better understand influence of the laser upon the surface chemistry of the glass, we performed XPS spectroscopic analysis on untreated and laser-treated glass samples. Fig. 4(b and c) show the O 1s spectra for the untreated and laser-treated glass, respectively. A comparison between the two reveals an additional oxidation peak in the laser-treated sample, corresponding to Si–(OH)_*x*_ groups (at 531 eV).^35^ Such increased concentration of hydrophilic species on the surface are expected to contribute to the increased hydrophilicity of the glass after laser treatment.

### Particles-separation/filtration

**Fig. 5:**
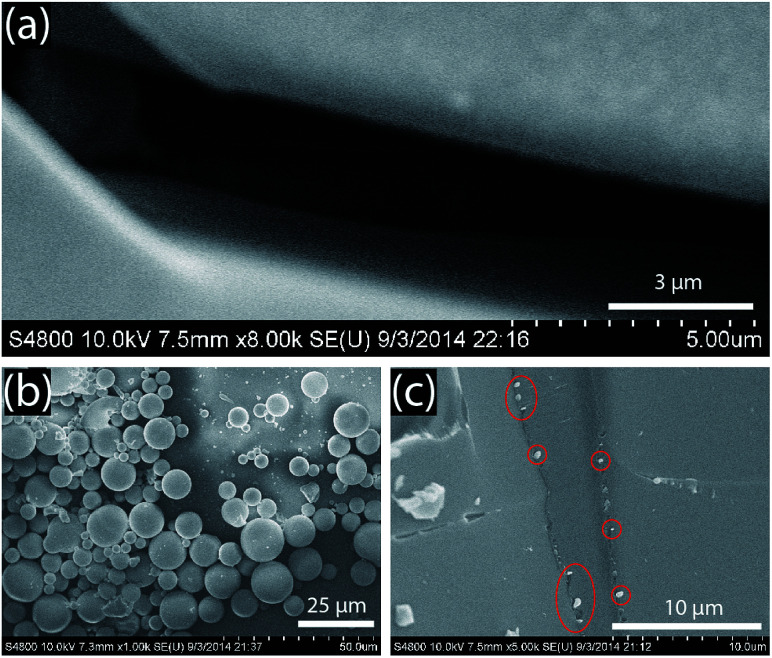
Cracked glass microchannel details. (a) Example of a 3–4 μm crevice. (b) Various particles of sizes 200 nm–13 μm at the inlet of the channel. (c) The end of the channel shows that the largest particles which can traverse the glass cracks are in the size range <3–4 μm.

The surfaces of the glass channels were observed using SEM imaging. Fig. 5(a) highlights a typical large cracked crevice on the channels with a width of about 3–4 μm, sufficiently small to filter out suspension components larger than 4 μm (*e.g.*, blood cells). The separation capability of cracked micro-channels was confirmed by loading the channel with an aqueous suspension containing a mixture of particles (*i.e.*, glass microspheres, iron oxide micro-particles, silver nanoparticles) of various sizes (200 nm–13 μm) and allowing the liquid to wick through the channel. After wicking was complete, the liquid was allowed to dry, and the sample was imaged to compare the beginning and end of the channel. As Fig. 5(b) shows, the glass structure retained larger particles (>4 μm) at the beginning, allowing only those smaller than 3–4 μm to reach the end of the channel, Fig. 5(c).

**Fig. 6:**
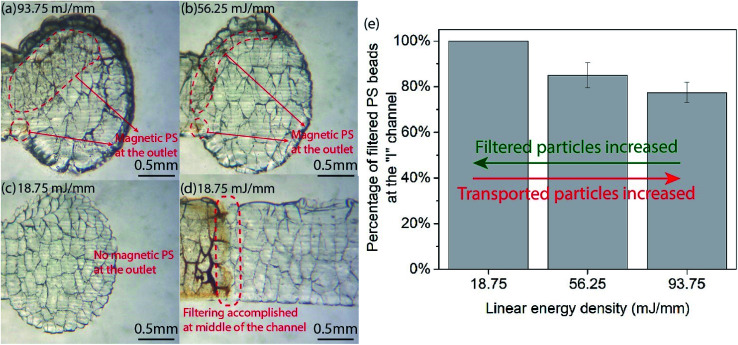
The quantitative filtering characterization of the laser-processed glass platform serving as a filtering mesh under different linear energy density. (a–d) Optical microscope images of magnetic PS particles (∼1.5 μm diameter) reaching at the outlet after traveling the “I” channel showing an inverse proportional relationship between the laser intensity and the filtering capability. When reducing the linear energy density from 93.75 to 18.75 mJ mm^−1^, the transported particles at the outlet are decreased, thus indicating an increase of filtered particles in the channel. (e) Analysis of PS beads filtered by the channel. The result demonstrates a compromised filtering capability in channels made using high laser intensity, compared to those created with lower intensity. 100% PS beads were filtered in the 18.75 mJ mm^−1^ channel, but only 87% and 77.5% were filtered for intensities of 56.25 and 93.75 mJ mm^−1^, respectively.

We also investigated the filtering performance in a quantitative manner using microchannel fabricated with different laser parameters. Fig. 6(a and b) show optical microscope images of polystyrene (PS) beads (∼1.5 μm diameter) reaching the end of a 20 mm long “I”-shaped channel. Decreasing the linear energy density from 93.75 to 56.25 mJ mm^−1^ results in a reduced number of the PS particles at the channel outlet. Moreover, at 18.75 mJ mm^−1^, a near complete filtering of PS was achieved with minimal transported PS found at the outlet, Fig. 6(c and d). The inverse proportional relationship between the laser intensity and the filtering capability was also verified by analyzing the scattered intensity of PS ratio between at the outlet and at the inlet. The result demonstrates a compromised filtering capability in channels made using high laser intensity, compared to those created with lower intensity. 100% PS beads were filtered in the 18.75 mJ mm^−1^ channel, but only 85% ± 5.5% and 77.5% ± 4.4% were filtered for intensities of 56.25 and 93.75 mJ mm^−1^, respectively, Fig. 6(e).

### Reproducibility and shelf life

For reproducibility evaluations, we fabricated a total of 42 devices under similar conditions, where 21 devices were tested at the same day and the other 21 ones were examined in 3 months in terms of shelf-life. The results of the former characterization show an average of 10.6 ± 1.5 mm s^−1^ (14.2% variation); whereas, the results of the latter present an average of 11.3 ± 2.9 mm s^−1^ (26% variation), an increased variation attributed to slight aging effects of storage. Overall, adding these two experiments together, we obtained a wicking speed of 10.8 ± 2.17 mm s^−1^, 20% variation, for a total of 42 samples. Therefore, the 20% variation of using these devices indicated that although the exact prediction of crack formations (geometry, size, and density) is physically impossible, the statistical average behavior of the fabricated platform is repeatable to within 80%.

In order to compare the reproducibility of the presented technique to that of filter paper, the most common wicking-driven substrate used in lab-on-a-chip applications, a similar experiment was conducted by wicking the Evans Blue solution at a constant pumping rate of 500 μL h^−1^ through 20 strips of 1.5 mm wide and 20 mm long filter paper (Whatman® qualitative filter paper, Grade 1). The results show an averaged wicking speed of 94.2 ± 14 μm s^−1^, with variation similar to the laser-induced cracked-glass (∼15%).

**Fig. 7:**
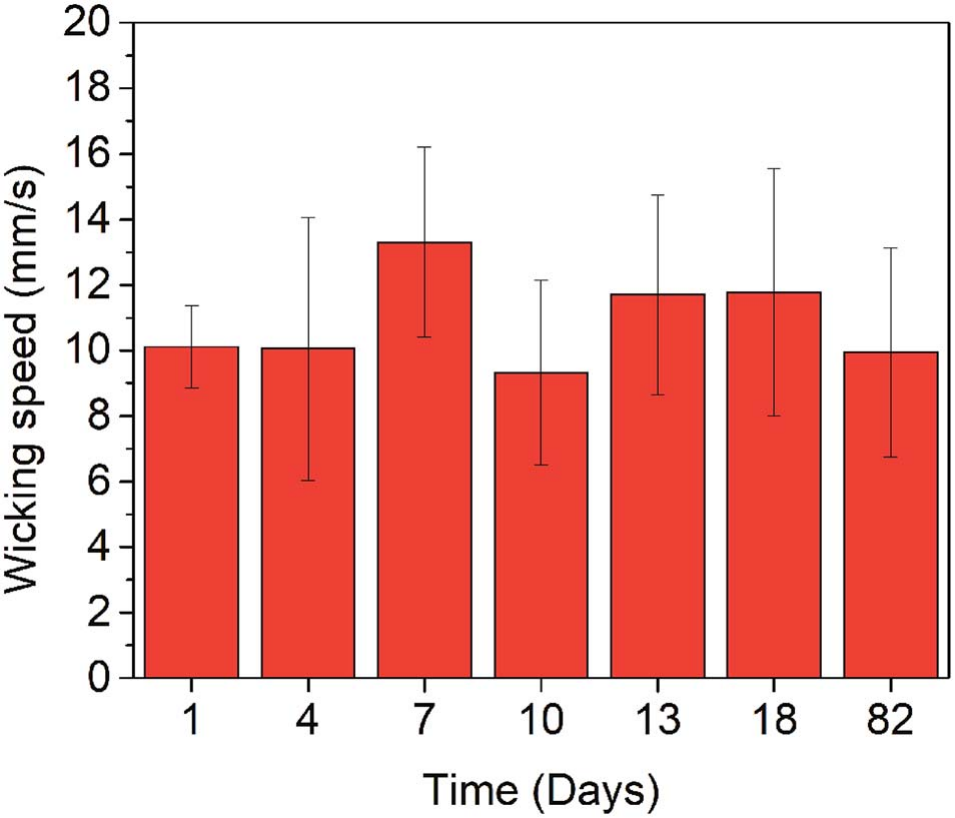
The shelf-life of the cracked-glass platform shows that the laser-created channels can maintain their initial performance in the range of 10–13 mm s^−1^ for up to 82 days. Each data point represents the average of 3 trials.

Fig. 7 presents the characterization results of the self-life. The platform was capable of transporting the liquid through the laser-ablated channels within the range of 10–13 mm s^−1^ (average of 11.3 ± 2.9 mm s^−1^) for up to 82 days (about 3 months). This wicking speed is close to the 13.6 ± 2.8 mm s^−1^ at day 0 (with an average of 17% performance decrease). It is reasonable to expect that the shelf-life (*i.e.*, long-term stability) of this laser-fabricated glass platform is at least close to 3 months under a normal storage condition.

## Conclusions

We presented a glass-based microfluidic platform based on CO_2_ laser machining of soda lime glass that combines high-speed wicking-driven liquid transport with size-based particle separation. This was done by controllably creating micro-cracks in the glass substrate by selectively raising the temperature above the strain point of the glass. The micro-crack formation and their pattern were investigated and correlated with the laser energy deposition rate. High energy levels resulted in large cracks (long crevices), a mode preferred to fabricate fast wicking speed microfluidic channels; while lower laser levels created small cracks (short crevices), favoured to form an enhanced particle-filtering mesh by increasing the cracks/sieves density. The process is as repeatable and can reproduce samples with 15% variations in wicking speed, comparable to commercial filter paper.

## Conflicts of interest

There are no conflicts to declare.

## Supplementary Material
